# How generative AI is shaping research software development and maintenance at a research-intensive university

**DOI:** 10.12688/openreseurope.22009.1

**Published:** 2026-02-23

**Authors:** Stephanie A. Besser, Eric A. Jensen, Daniel S. Katz

**Affiliations:** 1 School of Information Sciences, University of Illinois Urbana-Champaign, Urbana, Illinois, USA; 2 Institute for Methods Innovation, Dublin, Ireland; 3 University of Illinois Urbana-Champaign, Urbana, Illinois, USA

**Keywords:** generative AI, research software, software development, academic research, higher education, survey research, gender disparities, code generation, debugging, artificial intelligence adoption, research productivity, software maintenance, mixed methods, demographic patterns, research workflows, computational research, AI-assisted programming, research-intensive university, scholarly software, technology adoption

## Abstract

**Background:**

Generative artificial intelligence is spreading rapidly across academic research, yet its role in the development and maintenance of research software remains insufficiently characterized.

**Methods:**

A six week, institutional review board approved, anonymized online survey of faculty and research staff at a large research intensive university in late 2024 (n = 251). Branching survey questions distinguished general users of research software from those who create or maintain it. Quantitative associations were examined using chi square or Fisher’s exact tests, and free text descriptions of generative AI use in software development were analyzed thematically.

**Results:**

Overall, 29% of respondents reported using generative AI for at least one research task. Within the subsample of active research software developers, 33% reported using generative AI for software development and 51% indicated continued or planned future use. No statistically significant associations were found for age, recency of highest degree, or external funding. Gender was significantly associated with generative AI use for software development, with higher uptake among men than women (41% versus 15%; χ ^2^(1)=5.03, p=.025). Reported generative AI uses clustered around four practical roles: generating initial code and queries, supporting debugging and testing, transforming data or commands via natural language prompts, and reducing cognitive burden in repetitive or complex tasks.

**Conclusions:**

At a large research intensive university, generative AI adoption in research software development is already common among active developers and is expected to expand. The observed gender disparity signals a potential equity risk as tool assisted development becomes normalized. These findings provide an empirical baseline for multi institution replication and for evaluating how generative AI may reshape the organization and distribution of research software work.

## Introduction

The academic research process has always evolved as technology advances. For decades, research software has become an increasingly essential technology in many scholarly research fields. Research software comprises source code, algorithms, scripts, workflows and executables created during, or expressly for, the research process to collect, simulate, analyze, visualize or communicate scholarly data and findings as reported in 2021 ^1^. Research software plays a critical role in contemporary data sharing workflows and standards, as well as enabling the reuse of research data. While advances related to artificial intelligence (AI) have been affecting research processes for several decades, their role and significance is now more salient than ever with the ascendence of generative AI (GenAI) as studied in 2023 ^2^. In this study, we investigate the use of GenAI at the University of Illinois Urbana-Champaign, a research-intensive (R1) university, via an anonymized institutional survey, looking at overall patterns in GenAI research use and focusing specifically on research software.

### How researchers are using GenAI

Prior research in 2017 analyzing AI use in general almost a decade ago found AI was being used in academic research for a host of purposes, including surgical robotics, autonomous drones, experimental design, automated writing assessment, cancer diagnostics, and protein–protein interaction prediction ^3^. A 2023 study analyzing social media posts by researchers to examine how GenAI was being used revealed a focus on its utility in research implementation and publication, and a rapidly shifting landscape of researcher attitudes in favor of GenAI ^4^. Existing studies from 2023 to 2025 indicate that the ascendence of GenAI is vastly extending the range of research uses for AI quantitatively and qualitatively, with the effort both to leverage, and to mitigate the risks of, emerging GenAI capacities currently in full swing ^5– 9^.

Recent surveys from 2024 and 2025 reveal that GenAI tools are being rapidly adopted by academic researchers, though with varying degrees of effectiveness, enthusiasm, and caution ^10, 11^. A landmark survey published by Nature in 2023 collected responses from over 1,600 researchers globally, offering one of the broadest views of AI adoption in research to date ^12^. This survey, which targeted researchers who had recently published academic papers, revealed that approximately 30% of respondents were already using AI in their research, while 48% were directly involved in developing or researching AI technologies. Notably, only 4% of these researchers considered GenAI tools to be a “necessity” in their field, though about 26% believed these tools would become essential within the next decade. The survey found that researchers have been using GenAI for a wide range of tasks, including to create new study hypotheses and code, automate data acquisition, process new data, summarize, and write information from research articles during the research process.

Patterns of GenAI vary by research field, academic discipline and career stage. A survey supported by the Chan Zuckerberg Initiative (CZI) with a sample of biomedical researchers (n=770) in February-March 2024 found that 39% had used generative AI in their research with the intensity of use ranging from ‘rarely’ (15%) to ‘sometimes’ at (17%) and ‘regularly’ (7%) ^13^. In terms of specific research uses, this study also found that 22% had used GenAI to write code, 13% to create images or visualizations, and 12% to analyze large datasets ^13^.

In a 2024 survey of 60 faculty affiliate researchers at Michigan Institute for Data Science, researchers summarized the uses of GenAI using four categories: (1) text and image generation (including writing, compliance checks, using language for the general public), (2) disciplinary/interdisciplinary knowledge and insight collaboration, (3) all phases of the research process from project ideas and hypothesis generating to data analysis/visualization and report writing, and finally, (4) developing new research pathways, especially in sciences, such as protein structure models and aerospace engineering ^14^. This same study asked researchers, “How do you want to use GenAI in your research?”: 72% stated improving productivity, 63% said coding, 55% data analysis and modeling, 45% communication, and 38% helping with data generation processing and documentation ^14^. In addition, an international survey of digital humanities researchers (n=76) investigated how these researchers were using GenAI in their work, finding that GenAI was being used for creating presentations and helping to write grant applications, brainstorming ideas, writing code, and visualizing results ^15^. These digital humanities researchers also found benefits to using GenAI to increase research productivity by coding faster, assisting with language, and enhancing creativity ^15^.

In 2025, a large-scale survey of researchers in Denmark, from PhD students to full professors (n = 2,534), asked about using GenAI tools across the research stages, including project generation, study design development, data collection, data analysis, and reporting of findings ^16^. In the statistical analysis of demographic patterns in this study, no effects were identified. Still, there were significantly higher GenAI usage rates among early career researchers and those working in technical and quantitative sciences ^16^. A notable conclusion from this Danish study was “while reported use of GenAI is still fairly low, the reason for not engaging in more use cases of GenAI is probably not primarily related to research integrity considerations” ^16^. Moreover, this Danish study found a plurality of respondents considered GenAI to be an accelerator of research (40.7% of 1,032 researchers were positive about using GenAI for research design and data analysis), doing the research heavy lifting, and assisting with language during report writing ^16^.

A 2024 survey of researchers in Bahrain on GenAI literacy skills, awareness, usage, and concerns found that approximately 51% of 173 respondents used GenAI ^17^. The most common GenAI uses reported were writing and language support (49%), literature synthesis (15%), referencing support (9%), idea generation (6%), and data analysis (5%).

Despite the growing empirical literature on GenAI adoption in scholarly research, detailed information about how researchers across a range of academic disciplines use generative AI to develop research software is notably underdeveloped. To address this knowledge gap, we conducted a mixed methods survey of faculty and research staff at the University of Illinois Urbana-Champaign to measure GenAI usage relating to research software.

## Methods

We designed and implemented an online survey to examine the use and development of research software at the University of Illinois Urbana-Champaign, which included a set of questions pertaining to GenAI usage. The study proposal was reviewed and approved by the University of Illinois Urbana-Champaign Institutional Review Board (IRB Protocol: Project IRB24-0989). The survey was open for six weeks from late August-October 2024. All identifiable respondents' details were removed before analysis. The resulting anonymized dataset underpinning this article has been published on Zenodo, along with the full survey instrument used for the study ^18^.

### Survey design

The questionnaire opened with an informed consent statement outlining the study’s purpose, potential benefits and risks, voluntary participation and withdrawal rights, confidentiality safeguards, data security measures, estimated completion time, and investigator contact details. The instrument comprised four modules: (1) demographics, (2) use of software in research, (3) creation or maintenance of research software, and (4) citation practices for software. Branching logic terminated the survey at two points when subsequent sections were not relevant to a respondent based on their prior responses. This produced section-specific sample sizes, which were further differentiated when some participants chose not to respond to a given item or exit the survey at an unexpected point. As long as at least one outcome variable was completed by a respondent, their data were retained in the analysis. The vast majority of survey items were closed-ended, that is, single-choice, multiple-choice (i.e., “select all that apply”), and Likert-type, with a small number of open-ended questions. The survey design employed previously validated survey item scale categories ( Table 1) ^19^. Here, we focus exclusively on results relating to GenAI, with other results reported elsewhere due to length constraints.

**Table 1.** Survey Category Scales.

Scale	Categories
Frequency Scale	1-Not at all 2-Slightly 3-Somewhat 4-Moderately 5-Extremely 6-Not Applicable
Level of Quality	1-Poor 2-Fair 3-Good 4-Very Good 5-Excellent
Amount of Use Scale	1-None 2-Almost none 3-A small amount 4-A moderate amount 5-A great deal
Frequency – 5 Point Scale	1-Never 2-Rarely 3-Sometimes 4-Often 5-Always

### Sampling

The study adopted a convenience-snowball sampling approach. Initial e-mail invitations were sent to the heads of every department, center, and institute at the University of Illinois Urbana–Champaign. These invitations asked each person receiving the invitation to forward the questionnaire to their research personnel. Additional recruitment relied on one announcement in the campus Faculty and Staff Newsletter, a notice in the Social and Behavioral Sciences Bulletin, and recurrent posts in the National Center for Supercomputing Applications’ bi-weekly *Bytes and Pieces* newsletter. This sampling approach means the downstream distribution list, and thus the final sampling frame, cannot be determined. This multi-channel strategy offered a cost-efficient route to reach a heterogeneous pool of researchers across the institution, encompassing both users and non-users of research software as well as investigators who actively develop or maintain such software. However, this approach also brings risks of systematic sampling bias, including the possibility that those with no interest or involvement in research software would be less likely to respond.

**Figure 1.**
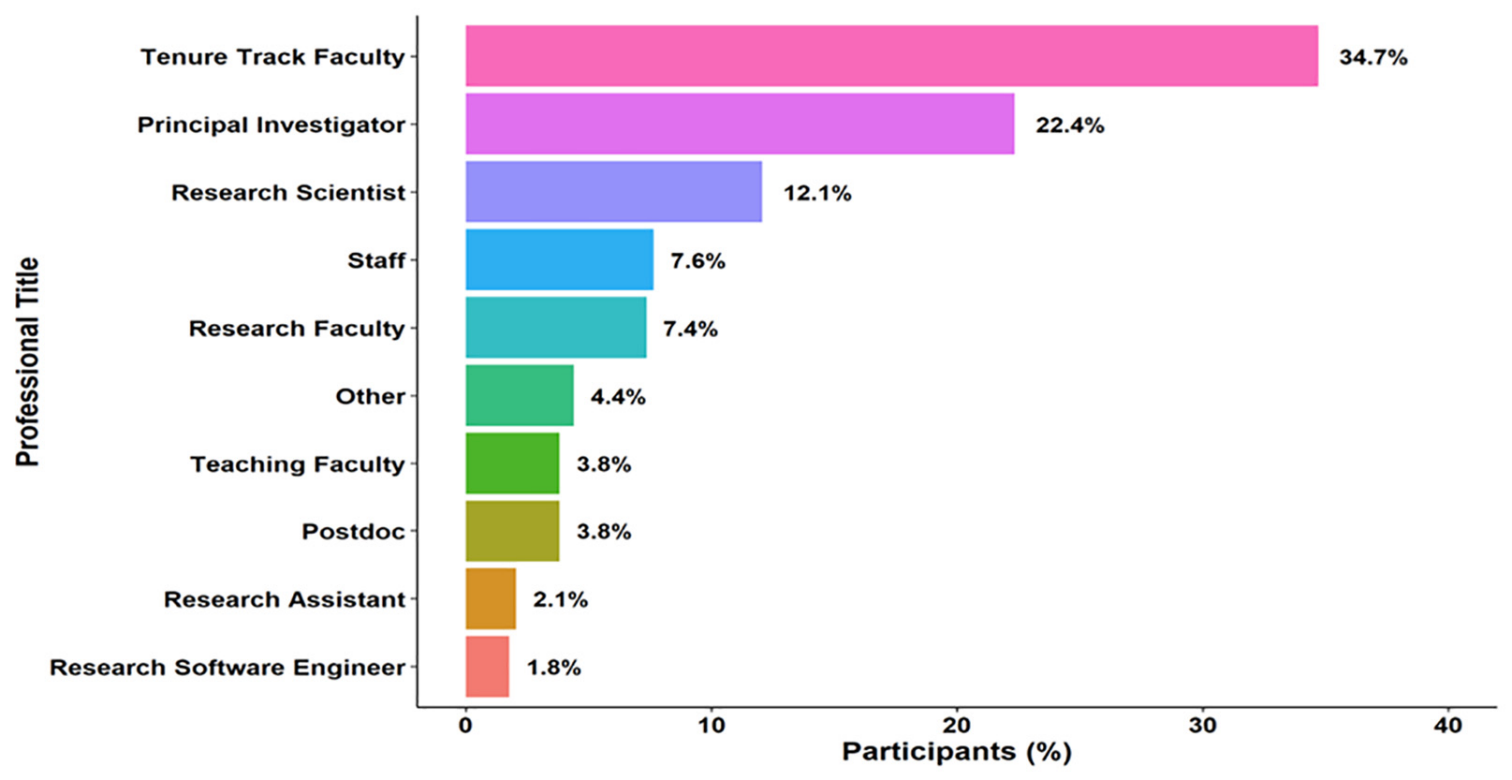
Survey respondents’ professional titles. Distribution of professional roles among survey respondents at a research-intensive university. Respondents could select multiple roles. Data were collected via an online survey administered between August and October 2024. The sample included faculty, principal investigators, research scientists and other research staff (n = 251 total respondents; total responses = 340 due to multiple selections). Bars represent the frequency of selections per role. Percentages are calculated relative to total respondents. No inferential statistics were applied.

**Figure 2.**
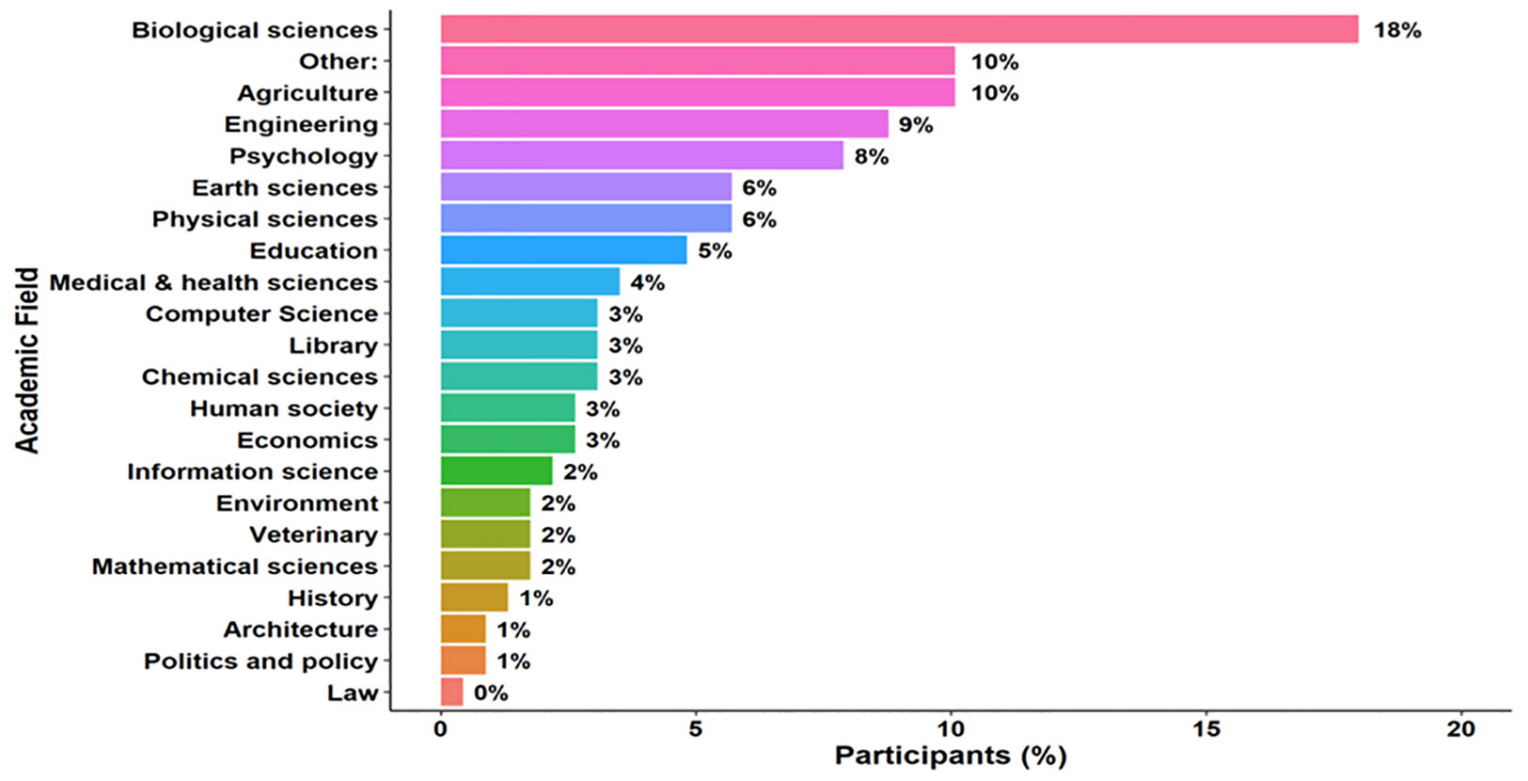
Survey respondents’ academic fields. Self-reported primary academic field of respondents who selected a single disciplinary category. Data derive from an online survey of faculty and research staff conducted in 2024. The figure summarizes responses from 228 participants who reported one field. Bars indicate the proportion of respondents in each field. Percentages are calculated within the single-response subsample.

***Survey respondents’ profiles***. A plurality of participants in a ‘select all that apply’ question identified as tenure-track faculty (35%, f = 118). Other common roles were principal investigator (22%, f = 76) and research scientist (12%, f = 41) ( Figure 1). Among the 228 respondents who specified a single disciplinary area, 18% were in biological sciences (f = 41), a combined total of 16% (f = 36) were in engineering, computer science, information science, mathematics and 10% were in agriculture (f = 23). Earth, physical, chemical, environmental, and veterinary sciences together represented 18% (f = 41). Remaining fields included education (5%, f = 11), economics (3%, f = 6), medical sciences (4%, f = 8), and politics/policy/law (1%, f = 3) (for a more specific Figure 2).

### Data management and analysis

A total of 256 individuals submitted responses. Four were removed for incomplete consent, and one graduate student—outside the target population of post-doctoral researchers, research staff, and faculty—was excluded, leaving 251 analyzable cases. The instrument contained two key branching (“stop”) questions, so the denominator or sample base varies from item to item. First, after demographics, respondents were asked whether they use research software; 39 answered “no” and exited at this stage, yielding a research software-user subsample of 212. A second filter question later in the survey asked whether respondents actively create or maintain research software; 138 said “no,” producing a subsample of 74 for the questions relating to creation of research software.

Responses such as *Not Applicable* and *Unsure* were used for descriptive statistics, but these responses were excluded from inferential statistical analyses. Prior to completing the survey, respondents were provided with a written consent form for their information to be used as part of research publication and anonymized public data. Participation required confirmation via this written consent form.

Because this branching survey structure resulted in sparsely populated contingency tables for some of the detailed, contingent survey items, response options were systematically collapsed to satisfy inferential statistical test assumptions by increasing expected values in contingency tables. Age was dichotomized (< 45 vs. ≥ 45), mirroring prior research ^20, 21^. Years since highest degree were recoded (< 10 years vs. ≥ 10 years), and gender was reduced to man versus woman because the non-binary gender count was too small for statistical estimation. No additional cases were removed for item non-response: respondents who did not reach a section had their non-response coded as missing data. This meant that the sample base for each statistical analysis could be different, and there was considerable variation from early-stage survey items that were more broadly applicable to the later stage, more specific items that were only applicable to a subset of the respondents.

Associations between demographic covariates and three binary outcomes—previous use of generative AI (GenAI) in any research task, use of GenAI in research software development, and intention to continue using GenAI for research software work—were analyzed with chi-square (χ ^2^) tests or, when expected cell sizes fell below five, Fisher’s exact tests. Results are reported with odds ratios and 95% confidence intervals; significant effects include Cramer’s V as an effect-size estimate.

### Data availability

Survey resources, including deidentified data, are available on Zenodo ^18^ and are freely accessible. The repository entry contains:

1. Original Survey in PDF format

2. Anonymized Survey Responses

3. REDCap Survey Codebook

## Results

**Figure 3.**
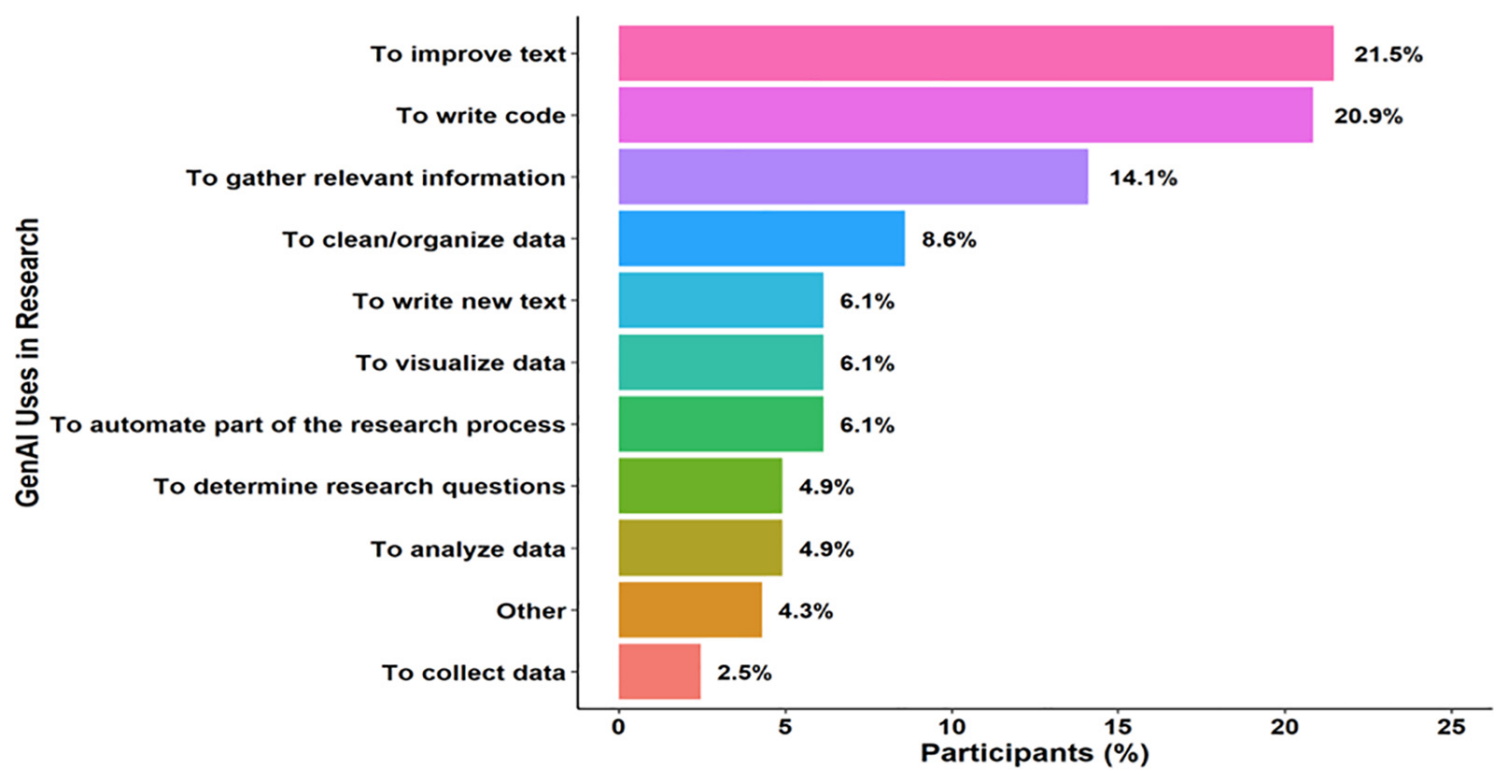
Uses of generative AI in research. Reported applications of generative artificial intelligence in research workflows among respondents who indicated prior GenAI use. Participants could select multiple uses. Data reflect responses from 163 unique researchers. Categories include text refinement, code generation, rapid information retrieval and other tasks. Bars represent the percentage of GenAI users reporting each application. Percentages exceed 100% in aggregate due to multiple responses per participant.

Twenty-nine percent (n= 61) of surveyed researchers reported integrating GenAI into their research workflows. Among these GenAI users, the leading applications were text refinement (22%, n = 35), code generation (21%, n = 34), and rapid information retrieval (14%, n = 23) ( Figure 3). Generative AI uptake for research software development lagged general research use: only 33% (n = 24) of respondents developing or maintaining software had adopted AI for that purpose. Nonetheless, a narrow majority (51%, n = 37) indicated they will continue—or plan to begin—to employ generative AI in future software-development tasks.

Age was not a statistically significant factor. Twenty-six per cent of respondents older than 45 years (n = 26) and 32% of those 45 years or younger (n = 34) reported using GenAI in their research, χ ^2^(1) = 0.92, p = .337 ( Table 2). Uptake of GenAI for research software development showed a similar non-significant pattern for age, with an adoption rate of 25% (n = 9) among older respondents versus 42% (n = 15) for younger respondents, χ ^2^(1) = 2.25, p = .134. Intentions to continue using GenAI in research software development were also statistically similar for older (49%, n = 18) and younger (54%, n = 19) respondents, χ ^2^(1) = 0.23, p = .632.

**Table 2.** GenAI Results by Age.

Characteristic	45 Years or Older	under 45 years old	Test Statistic	P-value
**Using GenAI in research**			Χ ^2^(1)=0.920	p=0.337
Yes	26/100 (26%)	34/106 (32%)		
No	74/100 (74%)	72/106 (68%)		
**Using GenAI in research** ** software development**			Χ ^2^(1)=2.250	p=0.134
Yes	9/36 (25%)	15/36 (42%)		
No	27/36 (75%)	21/36 (58%)		
**Continue to use GenAI** ** in research software** ** development**			Χ ^2^(1)=0.229	p=0.632
Yes	18/37 (49%)	19/35 (54%)		
No	19/37 (51%)	16/35 (46%)		

Statistically significant gender effects were not evident for general use of GenAI for research purposes: Adoption did not differ between men (33%, n = 31) and women (22%, n = 23), χ ^2^(1) = 2.96, p = .085 ( Table 3). However, men (41%, n = 17) were significantly more likely to employ GenAI for research software development than women (15%, n = 4), χ ^2^(1) = 5.03, p = .025, Cramer’s V = 0.24. Gender differences in intentions to continue using GenAI in research software development in the future did not reach the threshold for statistical significance (56% men versus 36% women, χ ^2^(1) = 2.49, p = .115).

**Table 3.** GenAI Usage Results by Gender.

Characteristic	Man	Woman	Test Statistic	P-value	Effect size
**Using GenAI in research**			Χ ^2^(1)=2.964	p=0.085	
Yes	31/93 (33%)	23/103 (22%)			
No	62/93 (67%)	80/103 (78%)			
**Using GenAI in research** ** software development**			**Χ ^2^(1)=5.028**	**p=0.025**	**Cramer’s V=0.241**
Yes	17/41 (41%)	4/26 (15%)			
No	24/41 (59%)	22/26 (85%)			
**Continue to use GenAI** ** in research software** ** development**			Χ ^2^(1)=2.485	p=0.115	
Yes	24/43 (56%)	9/25 (36%)			
No	19/43 (44%)	16/25 (64%)			

Recency of highest degree, as an indicator of level of research experience, was not a statistically significant factor. Among those whose terminal degree was awarded more than ten years ago, 25% (n = 33) reported GenAI research use compared with 36% (n = 28) of more recent graduates, χ ^2^(1) = 2.92, p = .087 ( Table 4). GenAI use for research software development was the same for both categories (33 %; χ ^2^ ≈ 0, p = 1.00), and plans to continue using GenAI for research software development were similar (50% for over 10 years versus 54% for under 10 years post-terminal degree), χ ^2^(1) = 0.11, p = .739).

**Table 4.** GenAI Usage Results by Last Degree Earned within 10 Years.

Characteristic	Last Degree Earned More than 10 Years Ago	Last Degree Earned Less than 10 Years Ago	Test Statistic	P-value
**Using GenAI in research**			Χ ^2^(1)=2.921	p=0.087
Yes	33/131 (25%)	28/77 (36%)		
No	98/131 (75%)	49/77 (64%)		
**Using GenAI in research ** **software development**			Χ ^2^(1)=0	p=1.000
Yes	16/48 (33%)	8/24 (33%)		
No	32/48 (67%)	16/24 (67%)		
**Continue to use GenAI** ** in research software ** **development**			Χ ^2^(1)=0.111	p=0.739
Yes	24/48 (50%)	13/24 (54%)		
No	24/48 (50%)	11/24 (46%)		

Respondents were also asked whether they receive external funding for their research. There was no statistically significant difference between those who do or do not receive such funding support. 31% (n = 49) of funded researchers reported using GenAI for research versus 24% (n = 12) of unfunded respondents, χ ^2^(1) = 0.81, p = .368 ( Table 5). Rates of GenAI use for research software development were also similar for these two categories (33% funded versus 38% unfunded; Fisher’s exact p = 1.00, OR = 0.82, 95% CI [0.14, 5.76]). The differences likewise did not reach the threshold for statistical significance in terms of the proportion planning to continue using GenAI in their research software development (48% versus 86%; Fisher’s exact p = .108, OR = 0.16, 95% CI [0.003, 1.39]).

**Table 5.** GenAI Results by Received Funding.

Characteristic	Received Funding	No Funding	Test Statistic	P-value
**Using GenAI in research**			Χ ^2^(1)=0.811	p=0.368
Yes	49/160 (31%)	12/50 (24%)		
No	111/160 (69%)	38/50 (76%)		
**Using GenAI in research** ** software development**			Fisher’s exact test, [OR 0.816, 95% CI, 0.143-5.757]	p=1.000
Yes	21/64 (33%)	3/8 (38%)		
No	43/64 (67%)	5/8 (62%)		
**Continue to use GenAI** ** in research software** ** development**			Fisher’s exact test, [OR 0.155, 95% CI, 0.003-1.391]	p=0.108
Yes	31/65 (48%)	6/7 (86%)		
No	34/65 (52%)	1/7 (14%)		

### How GenAI is used in software development

A follow-up open-ended question was posed to those who answered *yes* to the question, “Have you used generative AI in your software development?”. Responses to this question, “How has generative AI software assisted you in creating software?”, were analyzed and categorized ( Table 6).

**Table 6.** How researchers are using GenAI for software development.

GenAI software development use	Focus for this use	Illustrative data extracts
*AI-enhanced * *development*	Rapid generation of initial code skeletons, code, and queries based on either natural language or existing code, which developers later refine.	“Stubbed SQL queries, stubbed functions, proposed algorithms, became components in our pipelines.” “Initial code generation that is corrected and modified by an expert.” “AI tools like ChatGPT and Claude greatly speed up software development by producing useful pieces of code based on natural language prompts.” “It can provide a rough starting place for larger programs.” “I find it is terrible at creating something from nothing, but if I give it the code I am working on and what the goals are, then the output is usually 90 % of the way there and I just need to tweak a few things.”
*Streamlined debugging*	Faster fault localization, explanation, and automated test creation.	“Sometimes faster to type in some code in ChatGPT and ask what is wrong, than to debug.” “Code analysis/debugging.” “The AI tools also help with debugging existing code and producing unit tests.” “It’s good for quickly identifying solutions to specific error messages.”
*Natural language data* * transformation*	Using human language prompts rather than formal specifications to execute complex manipulations or retrieve commands	“Very useful for data manipulation or repeated operations which are difficult to math/logic describe but easy verbally.” “Helping discover new ways of writing code, optimizing existing code, or as a quick reference for basic functions… easier to ask ChatGPT which functions to use than to look through package documentation.” “Also faster to ask for commands for some programs like VMD than looking in the user manual.”
*Cognitive offloading*	Reducing mental effort and making detail-heavy tasks more approachable.	“Require less mental concentration than the similar manual work.” “Make it psychologically easier to take on a software development task that is conceptually simple but annoyingly complex.”

The main themes of how GenAI is used in software development were accelerated scaffolding, streamlined debugging, natural-language data transformation and cognitive offloading. These responses show that some researchers view GenAI as a productivity amplifier.

## Discussion

The survey shows that one-third of respondents have already integrated GenAI into research software development, and just over half intend to continue or begin doing so. This research software-specific uptake slightly exceeds the 29 percent who use GenAI for general research tasks, suggesting that coding is emerging as a leading application area. A striking gender gap emerged in our study, with male researchers nearly three times more likely than female colleagues to employ GenAI for research software-related uses. The most similar survey data on this topic internationally has not yet detected such a gender difference in GenAI adoption, suggesting that local academic cultures and specific GenAI use cases may yield different adoption patterns.

Open-ended responses illuminate how GenAI is being woven into workflows. Researchers reported using GenAI for rapid scaffolding of code, streamlined debugging, natural-language data transformation, and general cognitive offloading of repetitive programming tasks. These accounts portray GenAI as a productivity amplifier that accelerates early development stages while requiring human oversight for validation and refinement. While it is not yet clear whether these are the settled use cases for GenAI in research software, these offer a starting point or benchmark for future research to test against.

Several study limitations are worth highlighting at this stage. The study draws on a single institution and a self-selected sample, limiting generalizability. Branching logic reduced denominators for some analyses, and self-reporting introduces recall and desirability biases. Details such as GenAI usage intensity, model choice, and long-term performance effects were beyond the scope of the present study. Replication across diverse institutions and disciplines, combined with longitudinal tracking, is needed to confirm prevalence, examine causal drivers of demographic differences, and quantify downstream impacts on research software quality and impact. Even with these caveats, this study establishes an early baseline for monitoring GenAI’s integration into research software-related processes.

Nevertheless, these findings carry important implications, including for diversity and inclusion. Without proactive support, existing gender disparities in software engineering could widen as GenAI tools become integral to research software development. Conversely, by lowering technical barriers, well-designed GenAI assistants could democratize participation in research software development and bolster documentation standards.

## Ethics and consent

The study proposal was reviewed and approved by the University of Illinois Urbana-Champaign Institutional Review Board (IRB Protocol: Project IRB24-0989). Informed consent was obtained through a written online consent form prior to commencing the survey form. Details about how informed consent was gathered are provided with the underlying data published in Zenodo ^18^.

## Data Availability

Underlying and extended data supporting the findings of this work are available in Zenodo. Repository name: Research Software at the University of Illinois Urbana-Champaign: A Mixed Methods Survey Dataset. https://doi.org/10.5281/zenodo.15161372 ^18^. The project contains the following underlying data: Software_in_Research_Survey_Data.csv (raw, deidentified survey response data used for quantitative and mixed-methods analyses). Repository name: Research Software at the University of Illinois Urbana-Champaign: A Mixed Methods Survey Dataset. https://doi.org/10.5281/zenodo.15161372 ^18^. This project contains the following extended data: Research Software at the University of Illinois Survey.pdf (final formatted version of the questionnaire as presented to respondents). Research Software at the University of Illinois Survey Codebook.pdf (variable definitions, response options, and coding framework supporting interpretation and reuse of the survey data). Research Software at the University of Illinois Survey Consent Form.pdf (written participant information and consent documentation provided prior to survey participation). All underlying and extended data are available under the terms of the Creative Commons Attribution 4.0 International.
