# Supplementary material for: How generative AI is shaping research software development and maintenance at a research-intensive university
Source: Open Res Eur. 2026 Feb 23;6:56. [Version 1] doi: 10.12688/openreseurope.22009.1 (PMC13000399; doi:10.12688/openreseurope.22009.1)
Supplement: Peer review history

# Reviewer report: Reviewer response for version 1

**DOI:** 10.21956/openreseurope.23817.r70242

**Published:** 2026-03-18

**Authors:** Ludo Waltman

**Affiliations:** 1 Leiden University, Leiden, The Netherlands

This paper analyzes the role of generative AI in development and maintenance of research software. The authors surveyed researchers at University of Illinois Urbana–Champaign. The survey was carried out in August-October 2024, so the results may be somewhat outdated and may not reflect the current attitudes of researchers toward generative AI.

 Like many survey studies, this study has limitations related to a relatively small sample size and self-selection of survey respondents. The authors acknowledge the limitations of their survey. Despite these limitations, the results reported by the authors are helpful to better understand researcher attitudes toward generative AI. The paper is clearly written and the analyses carried out by the authors appear sound to me.

 As one of their main findings, the authors report that “gender was significantly associated with generative AI use for software development, with higher uptake among men than women”. The authors refer to this as a “striking gender gap” and suggest that “local academic cultures and specific GenAI use cases” may explain this gender gap. I believe some caution is warranted here. The authors found an association between gender and generative AI use, not a causal connection. There could be all kinds of confounding factors that may explain the observed gender difference. For instance, generative AI use may be more common in some disciplines than in others, and men may be overrepresented in the former disciplines and women in the latter ones.

If applicable, is the statistical analysis and its interpretation appropriate?

Partly

Is the study design appropriate and is the work technically sound?

Yes

Is the work clearly and accurately presented and does it engage with the current literature?

Yes

Are the conclusions drawn adequately supported by the results?

Partly

Are sufficient details of methods and analysis provided to allow replication by others?

Yes

Are all the source data and materials underlying the results available?

Yes

Reviewer Expertise:

scholarly communication, open science

I confirm that I have read this submission and believe that I have an appropriate level of expertise to confirm that it is of an acceptable scientific standard, however I have significant reservations, as outlined above.

# Reviewer report: Reviewer response for version 1

**DOI:** 10.21956/openreseurope.23817.r70244

**Published:** 2026-03-14

**Authors:** Gerasimos Chourdakis

**Affiliations:** 1 University of Stuttgart,, Stuttgart, Germany

This manuscript presents the results of a 2024 survey among researchers at the University of Illinois Urbana-Champaign regarding research software, focusing on the use of GenAI among developers of such research software. The manuscript relates the use of AI in research and research software development to demographics (age, gender, recency of highest degree, external funding), observing a gender gap in use of GenAI for research software development (41% for men vs 15% for women) and no significance of the other factors. Overall, 251 responses across disciplines were considered in the analysis, of which 74 referring to creating research software. The results are extracted from a survey with more questions around the use and development of research software.

 The paper has a clear research question, it is of appropriate length, easy to read, and the analysis and results are presented mostly clearly. At parts, I get the impression that further analysis and relation to literature could better support/discuss the findings.

 The gender gap is an interesting finding, especially since the data is specific to research software development, where literature seems to indeed be lacking. The paper claims that "The most similar survey data on this topic internationally has not yet detected such a gender difference in GenAI adoption, suggesting that local academic cultures and specific GenAI use cases may yield different adoption patterns" (unclear which one is "the most similar" reference - cite again). Since this is the most interesting finding, I would expect some comparison to the literature. Even if literature for research software is not available, it would be interesting to compare with GenAI adoption in other fields or a broader survey on use of GenAI in software engineering or research (Google Scholar Labs, for example, returns a few such references when asked "Is there a gender gap in adoption of GenAI tools in software engineering?"). The supplementary data of the cited reference 12 could also be used for a comparison: the ratio of men:women in each category seems to be "studyAI": 52%:38%, "useAI": 28%:36%, "notuseAI": 20%:26%.

 I also think that, since the sample is not from one discipline, the analysis should take into account that the ratio of men:women might be different among disciplines that show different adoption rates of GenAI.

 Further points that could strengthen the manuscript:

 - The survey includes 251 reponses. What fraction of the university staff is that? How representative is the sample to the population of the university (field, gender, ...)?

 - In some places, it is not immediately clear how the number of total responses is defined from the filtering of total responses. In particular, in Figure 3, the number of "163 unique researchers" is not immediately clear from the description in the text directly above, or from the different tables.

 - The free text responses on how AI is used seem to be 19, looking at the raw data. This number would be useful context for Table 6.

 - The survey is from summer 2024, but the use cases, mode of use (e.g., agents), and adoption keep evolving. What relevant changes should a next iteration of such a survey consider?

 - The International RSE survey 2026 is currently running and includes similar questions (the latest iteration from 2022 did not). Results might be interesting to compare to, if available in time: https://softwaresaved.limequery.com/611257

If applicable, is the statistical analysis and its interpretation appropriate?

I cannot comment. A qualified statistician is required.

Is the study design appropriate and is the work technically sound?

Partly

Is the work clearly and accurately presented and does it engage with the current literature?

Partly

Are the conclusions drawn adequately supported by the results?

Partly

Are sufficient details of methods and analysis provided to allow replication by others?

Yes

Are all the source data and materials underlying the results available?

Yes

Reviewer Expertise:

I am experienced in developing research software in academia (doing research on simulation software), I am exposed to GenAI tools for this purpose, and I am involved in the German RSE community.

I confirm that I have read this submission and believe that I have an appropriate level of expertise to confirm that it is of an acceptable scientific standard, however I have significant reservations, as outlined above.

# Reviewer report: Reviewer response for version 1

**DOI:** 10.21956/openreseurope.23817.r70247

**Published:** 2026-03-12

**Authors:** Siamak Farshidi

**Affiliations:** 1 Information Technology, Wageningen University & Research (Ringgold ID: 4508), Wageningen, Gelderland, The Netherlands

**Summary of the article:**

 This manuscript reports the results of a mixed methods survey examining how generative artificial intelligence (GenAI) is used in research software development at a research intensive university. The authors conducted a six week online survey of faculty and research staff at the University of Illinois Urbana Champaign and obtained 251 usable responses. The survey distinguishes between general users of research software and respondents who actively create or maintain research software. Quantitative analyses examine associations between demographic variables such as age, gender, years since highest degree, and external funding and three outcomes: use of GenAI in research, use of GenAI in research software development, and intention to continue using GenAI in software development. The statistical analysis relies primarily on chi square tests or Fisher’s exact tests. In addition, open ended responses describing how GenAI tools assist with software development are grouped into thematic categories such as code generation, debugging, natural language data manipulation, and cognitive offloading. 

 The results suggest that about 29 percent of respondents report using generative AI for research tasks and that among respondents who develop or maintain research software, 33 percent report using GenAI for software development tasks. The authors report no statistically significant associations between GenAI adoption and age, years since highest degree, or external funding. However, they report a statistically significant gender difference in GenAI use for software development, with higher reported adoption among men than women. The qualitative responses are used to illustrate typical uses of GenAI in programming workflows. The authors conclude that GenAI is already being integrated into research software workflows and that demographic differences in adoption could have implications for equity as AI assisted development becomes more widespread.

 The manuscript addresses a timely topic and provides useful descriptive information about how researchers are beginning to incorporate generative AI into software development workflows. However, several methodological and analytical limitations affect the strength of the conclusions. While the study can provide exploratory insights, some of the claims, particularly regarding demographic differences, are stronger than what the data can reliably support.

**Major comments:**

 The study relies on a convenience and snowball sampling strategy distributed through department heads and institutional newsletters. Because the final distribution list is unknown, the sampling frame and response rate cannot be determined. This has important implications. The respondents are likely self selected, especially individuals already interested in software or AI tools. The results therefore cannot be interpreted as population estimates of GenAI use among researchers. Comparisons across demographic groups may also reflect selection effects rather than real differences in adoption. The authors acknowledge some of these limitations, but the implications for interpretation should be emphasized more clearly. The findings should be framed explicitly as exploratory and descriptive rather than representative estimates of adoption.

 A second issue concerns the small analytic subsamples used for the key statistical analyses. Although the overall sample includes 251 respondents, the analyses of software development behavior rely on the subset of respondents who actively create or maintain research software. After applying the survey branching logic, this group is relatively small and the demographic comparisons rely on even smaller subgroups. In particular, the reported gender difference in GenAI use for software development is based on a small number of individuals reporting use in the relevant subgroup. Estimates based on such small cell counts can be unstable and sensitive to small changes in the data. The authors should report confidence intervals for the relevant proportions and effect sizes and should explicitly acknowledge the limited statistical power of the demographic analyses. The gender difference should be framed as a tentative observation rather than a robust finding.

 The analysis also involves multiple statistical tests across several demographic predictors and outcomes. However, the manuscript does not address the issue of multiple comparisons. Conducting many hypothesis tests increases the probability that statistically significant findings arise by chance. The authors should either apply an appropriate correction for multiple testing or clearly state that the analyses are exploratory and that the reported p values should be interpreted cautiously in that context.

 Another limitation of the statistical analysis is that it relies exclusively on bivariate tests. While chi square and Fisher’s exact tests are appropriate for categorical data, they do not control for potential confounding variables. Differences in GenAI adoption could plausibly be associated with disciplinary field, research role, or level of programming experience. Without multivariate analysis it is difficult to interpret whether the observed demographic differences reflect independent associations or underlying structural differences between groups. If feasible, the authors could strengthen the analysis by estimating a simple logistic regression model predicting GenAI adoption that includes several demographic and professional variables as covariates.

 The manuscript also includes qualitative responses categorized into themes such as accelerated development, debugging assistance, natural language transformation, and cognitive offloading. These examples are informative but the methodological description of the qualitative analysis is minimal. The paper does not explain how codes were generated, whether more than one coder was involved, whether any procedures were used to assess coding reliability, or how many responses correspond to each category. Providing a brief description of the qualitative coding procedure would improve transparency and reproducibility.

**Minor comments:**

 The decision to dichotomize variables such as age and years since degree reduces the information available in the data and may reduce statistical power. The authors should clarify the rationale for these thresholds or acknowledge the limitations of this approach. Because the survey uses branching logic, the effective sample size varies across analyses. It would improve clarity if the authors consistently reported the analytic sample size for each statistical comparison. The discussion could also engage more directly with literature on AI assisted programming, developer productivity tools, and gender disparities in computing, which would help situate the findings more strongly within existing research.

If applicable, is the statistical analysis and its interpretation appropriate?

Partly

Is the study design appropriate and is the work technically sound?

Partly

Is the work clearly and accurately presented and does it engage with the current literature?

Partly

Are the conclusions drawn adequately supported by the results?

Partly

Are sufficient details of methods and analysis provided to allow replication by others?

Partly

Are all the source data and materials underlying the results available?

Yes

Reviewer Expertise:

Software engineering and research software engineering, with expertise in AI for software engineering, empirical software engineering, and decision support systems for software technology selection. My research includes technology adoption in software ecosystems, evaluation of software platforms and tools, and AI driven decision making in research software environments and virtual research infrastructures.

I confirm that I have read this submission and believe that I have an appropriate level of expertise to state that I do not consider it to be of an acceptable scientific standard, for reasons outlined above.

# Reviewer report: Reviewer response for version 1

**DOI:** 10.21956/openreseurope.23817.r70245

**Published:** 2026-03-11

**Authors:** Akshay Mittal

**Affiliations:** 1 University of the Cumberlands, Williamsburg, KY, USA

**Executive Summary** This paper presents a timely, well-structured observational study investigating the adoption of generative artificial intelligence (GenAI) in research software development at a research-intensive university. By utilizing a six-week, IRB-approved survey with branching logic, the authors successfully distinguish general GenAI users from active research software developers. The study provides a methodologically sound snapshot of early GenAI adoption rates (33% among developers) and reveals a significant gender disparity in usage. Overall, the manuscript establishes a valuable empirical baseline for future research.

**Strengths**

- **Methodological Rigor:** The use of branching logic to accurately isolate active software creators/maintainers ensures the data is highly relevant to the research question.
- **Statistical Honesty:** The authors appropriately handled sparse contingency tables by collapsing categories to satisfy the assumptions of chi-square and Fisher's exact tests.
- **Open Science:** Making the deidentified dataset, REDCap codebook, and survey instrument freely available on Zenodo is highly commendable and ensures reproducibility.
- **Qualitative Insight:** The thematic analysis of open-ended responses effectively categorizes practical GenAI use cases into scaffolding, debugging, natural-language transformation, and cognitive offloading.

**Constructive Feedback & Minor Revisions:** While the paper is scientifically sound and should be approved, the authors could address the following points to strengthen the manuscript's impact:

1. **Contextualize the Gender Gap:** The discovery of a significant gender disparity (41% men vs. 15% women) is a critical finding. The discussion section would benefit from a brief expansion that hypothesizes why this gap exists, perhaps by drawing on broader literature on gender disparities in computer science or early technology adoption.
2. **Address Sample Size in Non-Significant Results:** For variables lacking statistical significance (e.g., age and funding), explicitly remind the reader in the discussion that the branching logic resulted in smaller subsamples for these specific tests. This prevents readers from over-interpreting the null result, as the study might simply be underpowered to detect smaller demographic effects.
3. **Propose Actionable Interventions:** The authors rightly note that existing gender disparities could widen without "proactive support." Providing 1-2 concrete examples of institutional interventions (e.g., targeted training workshops, equitable access to paid AI tiers) would increase the paper's practical utility for academic administrators.

If applicable, is the statistical analysis and its interpretation appropriate?

Yes

Is the study design appropriate and is the work technically sound?

Yes

Is the work clearly and accurately presented and does it engage with the current literature?

Yes

Are the conclusions drawn adequately supported by the results?

Yes

Are sufficient details of methods and analysis provided to allow replication by others?

Yes

Are all the source data and materials underlying the results available?

Yes

Reviewer Expertise:

Gen Ai, Cloud NAtive, ML

I confirm that I have read this submission and believe that I have an appropriate level of expertise to confirm that it is of an acceptable scientific standard.
